# (*E*)-4-[(1,5-Dimethyl-3-oxo-2-phenyl-2,3-dihydro-1*H*-pyrazol-4-yl)imino­meth­yl]phenyl 4-bromo­benzene­sulfonate

**DOI:** 10.1107/S1600536808035034

**Published:** 2008-10-31

**Authors:** Jian-Rong Han, Xia Tian, Xiao-Li Zhen, Zhen-Chao Li, Shou-Xin Liu

**Affiliations:** a College of Sciences, Hebei University of Science & Technology, Shijiazhuang 050018, People’s Republic of China; b College of Chemical & Pharmaceutical Engineering, Hebei University of Science & Technology, Shijiazhuang 050018, People’s Republic of China

## Abstract

In the title compound, C_24_H_20_BrN_3_O_4_S, the central benzene ring makes dihedral angles of 17.13 (13), 39.83 (13) and 58.37 (13)°, respectively, with the pyrazolone ring, the bromo­benzene ring and the terminal phenyl ring. In the crystal structure, the packing is stabilized by a weak non-classical inter­molecular C—H⋯O hydrogen bond which links the mol­ecules into a chain propagating in [100].

## Related literature

For a related structure, see: Han *et al.* (2007 ▶). For general background, see: Kahwa *et al.* (1986 ▶); Klayman *et al.* (1979 ▶); Santos *et al.* (2001 ▶). For reference geometrical data: see: Allen *et al.* (1987 ▶).
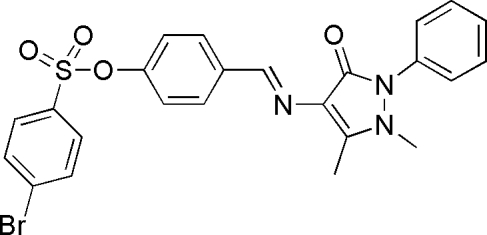


## Experimental

### 

#### Crystal data

C_24_H_20_BrN_3_O_4_S
*M* *_r_* = 526.40
Monoclinic, 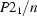

*a* = 6.9959 (14) Å
*b* = 33.222 (6) Å
*c* = 10.218 (2) Å
β = 95.992 (3)°
*V* = 2361.9 (8) Å^3^
*Z* = 4
Mo *K*α radiation
μ = 1.86 mm^−1^
*T* = 294 (2) K
0.18 × 0.16 × 0.11 mm

#### Data collection

Bruker SMART APEX CCD area-detector diffractometer
Absorption correction: multi-scan (*SADABS*; Sheldrick, 1996 ▶) *T*_min_ = 0.693, *T*_max_ = 0.815
12151 measured reflections
4174 independent reflections
2506 reflections with *I* > 2σ(*I*)
*R*_int_ = 0.058

#### Refinement

*R*[*F* ^2^ > 2σ(*F* ^2^)] = 0.044
*wR*(*F* ^2^) = 0.107
*S* = 1.01
4174 reflections
300 parameters
H-atom parameters constrained
Δρ_max_ = 0.28 e Å^−3^
Δρ_min_ = −0.23 e Å^−3^

### 

Data collection: *SMART* (Bruker, 1999 ▶); cell refinement: *SAINT* (Bruker, 1999 ▶); data reduction: *SAINT*; program(s) used to solve structure: *SHELXS97* (Sheldrick, 2008 ▶); program(s) used to refine structure: *SHELXL97* (Sheldrick, 2008 ▶); molecular graphics: *SHELXTL* (Sheldrick, 2008 ▶); software used to prepare material for publication: *SHELXTL*.

## Supplementary Material

Crystal structure: contains datablocks I, global. DOI: 10.1107/S1600536808035034/hb2828sup1.cif

Structure factors: contains datablocks I. DOI: 10.1107/S1600536808035034/hb2828Isup2.hkl

Additional supplementary materials:  crystallographic information; 3D view; checkCIF report

## Figures and Tables

**Table 1:** Hydrogen-bond geometry (Å, °)

*D*—H⋯*A*	*D*—H	H⋯*A*	*D*⋯*A*	*D*—H⋯*A*
C17—H17*A*⋯O4^i^	0.96	2.40	3.361 (5)	176

Symmetry code: (i) 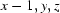.

**Fig. 1.**
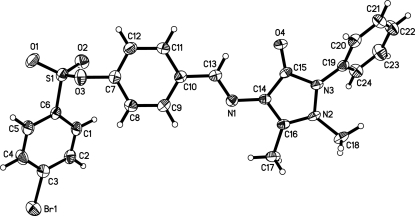
The molecular structure of (I) with displacement ellipsoids for non-H atoms drawn at the 30% probability level.

**Fig. 2.**
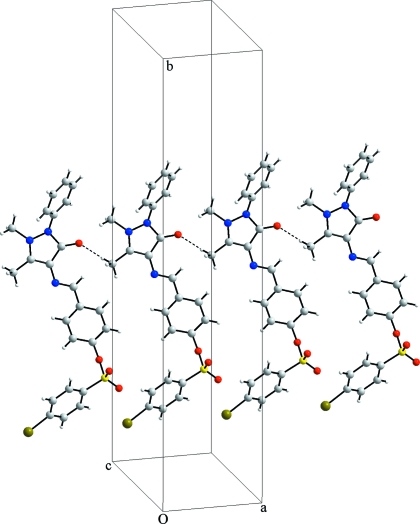
Packing diagram for (I), with H bonds drawn as dashed lines.

## supplementary crystallographic information

### Comment

The synthesis and structure of Schiff bases have attracted much attention in biology and chemistry (Kahwa *et al.*, 1986; Klayman *et al.*, 1979). Many Schiff base derivatives have been synthesized and employed to develop protein and enzyme mimics (Santos *et al.*, 2001). Among the large number of compounds, 4-amino-1,5-dimethyl-2-phenylpyrazol-3-one forms a variety of Schiff bases with aldehydes, and the synthesis and crystal structures of some of them, such as (*E*)-4-[(1,5-Dimethyl-3-oxo-2-phenyl-2,3-dihydro-1*H*-pyrazol-4-ylimino) methyl]-phenyl 4-chlorobenzoate (Han *et al.*, 2007) has been reported.

As part of an investigation of the potential coordination properties of Schiff bases that could function as ligands, we now report the synthesis and structure of the title compound, (I).

In the title molecule (Fig. 1), the pyrazolone ring (C14—C16/N1/N2/N3/O4) is nearly planar, with an r.m.s. deviation for fitted atoms of 0.029 Å. It makes a dihedral angle of 50.07 (13)° with its attached phenyl ring (C19—C24). The central benzene ring (C7—C13/O3) is almost planar, with an r.m.s. deviation for fitted atoms of 0.040Å. This group makes dihedral angles of 17.13 (13)°, 39.83 (13)° and 58.37 (13)°, respectively, with the the pyrazolone ring (C14—C16/N1/N2/N3/O4), the terminal C1—C6 benzene ring and the terminal C19—C24 phenyl ring. Otherwise, all bond lengths and angles are within their normal ranges (Allen *et al.*, 1987).

In the crystal, the packing is stabilized by a weak, non-classical intermolecular C17—H17A···O4 hydrogen bond that links molecules into one-dimensional extended chains running along the *a* axis (Table 1, Fig. 2).

### Experimental

An anhydrous ethanol solution (50 ml) of 4-formylphenyl 4-bromobenzenesulfonate (3.41 g, 10 mmol) was added to an anhydrous ethanol solution (50 ml) of 4-amino-1,5-dimethyl-2-phenylpyrazol-3-one (2.03 g, 10 mmol) and the mixture stirred at 350 K for 3 h under N_2_, giving a yellow precipitate. The product was isolated, recrystallized from acetonitrile, and then dried in a vacuum to give pure compound (I) in 87% yield. Yellow blocks of (I) were obtained by slow evaporation of an acetonitrile solution.

### Refinement

The H atoms were included in calculated positions (C—H = 0.93–0.96Å) and refined as riding with *U*_iso_(H) = 1.2*U*_eq_(C) or 1.5*U*_eq_(methyl C).

### Crystal data

**Table:**

C_24_H_20_BrN_3_O_4_S	*F*(000) = 1072
*M**_r_* = 526.40	*D*_x_ = 1.480 Mg m^−^^3^
Monoclinic, *P*2_1_/*n*	Mo *K*α radiation, λ = 0.71073 Å
Hall symbol: -P 2yn	Cell parameters from 1963 reflections
*a* = 6.9959 (14) Å	θ = 2.9–25.2°
*b* = 33.222 (6) Å	µ = 1.86 mm^−^^1^
*c* = 10.218 (2) Å	*T* = 294 K
β = 95.992 (3)°	Block, yellow
*V* = 2361.9 (8) Å^3^	0.18 × 0.16 × 0.11 mm
*Z* = 4	

### Data collection

**Table:**

Bruker SMART APEX CCD area-detector diffractometer	4174 independent reflections
Radiation source: fine-focus sealed tube	2506 reflections with *I* > 2σ(*I*)
graphite	*R*_int_ = 0.058
φ and ω scans	θ_max_ = 25.0°, θ_min_ = 2.1°
Absorption correction: multi-scan (SADABS; Sheldrick, 1996)	*h* = −8→8
*T*_min_ = 0.693, *T*_max_ = 0.815	*k* = −39→27
12151 measured reflections	*l* = −12→10

### Refinement

**Table:**

Refinement on *F*^2^	Secondary atom site location: difference Fourier map
Least-squares matrix: full	Hydrogen site location: inferred from neighbouring sites
*R*[*F*^2^ > 2σ(*F*^2^)] = 0.044	H-atom parameters constrained
*wR*(*F*^2^) = 0.107	*w* = 1/[σ^2^(*F*_o_^2^) + (0.0386*P*)^2^ + 1.1353*P*] where *P* = (*F*_o_^2^ + 2*F*_c_^2^)/3
*S* = 1.01	(Δ/σ)_max_ = 0.001
4174 reflections	Δρ_max_ = 0.28 e Å^−^^3^
300 parameters	Δρ_min_ = −0.23 e Å^−^^3^
0 restraints	Extinction correction: SHELXTL (Sheldrick, 2008), Fc^*^=kFc[1+0.001xFc^2^λ^3^/sin(2θ)]^-1/4^
Primary atom site location: structure-invariant direct methods	Extinction coefficient: 0.0031 (5)

### Special details

**Table:**

Geometry. All e.s.d.'s (except the e.s.d. in the dihedral angle between two l.s. planes) are estimated using the full covariance matrix. The cell e.s.d.'s are taken into account individually in the estimation of e.s.d.'s in distances, angles and torsion angles; correlations between e.s.d.'s in cell parameters are only used when they are defined by crystal symmetry. An approximate (isotropic) treatment of cell e.s.d.'s is used for estimating e.s.d.'s involving l.s. planes.
Refinement. Refinement of *F*^2^ against ALL reflections. The weighted *R*-factor *wR* and goodness of fit *S* are based on *F*^2^, conventional *R*-factors *R* are based on *F*, with *F* set to zero for negative *F*^2^. The threshold expression of *F*^2^ > 2σ(*F*^2^) is used only for calculating *R*-factors(gt) *etc*. and is not relevant to the choice of reflections for refinement. *R*-factors based on *F*^2^ are statistically about twice as large as those based on *F*, and *R*- factors based on ALL data will be even larger.

### Fractional atomic coordinates and isotropic or equivalent isotropic displacement parameters (Å^2^)

**Table:**

	*x*	*y*	*z*	*U*_iso_*/*U*_eq_	
Br1	−0.44278 (7)	0.221319 (15)	−0.16276 (5)	0.0730 (2)	
S1	0.32046 (15)	0.32822 (3)	−0.18138 (10)	0.0519 (3)	
N1	0.0354 (4)	0.49730 (9)	0.2384 (3)	0.0428 (8)	
N2	−0.1008 (4)	0.56730 (9)	0.4793 (3)	0.0424 (8)	
N3	0.0680 (4)	0.58680 (9)	0.4468 (3)	0.0413 (8)	
O1	0.4033 (4)	0.31657 (9)	−0.2973 (3)	0.0710 (9)	
O2	0.4292 (4)	0.32724 (8)	−0.0550 (3)	0.0649 (8)	
O3	0.2468 (4)	0.37319 (7)	−0.2123 (2)	0.0557 (7)	
O4	0.3126 (3)	0.57085 (8)	0.3161 (3)	0.0523 (7)	
C1	0.0241 (6)	0.29604 (12)	−0.0562 (4)	0.0516 (10)	
H1	0.0806	0.3080	0.0206	0.062*	
C2	−0.1397 (6)	0.27304 (12)	−0.0533 (4)	0.0580 (11)	
H2	−0.1926	0.2694	0.0257	0.070*	
C3	−0.2254 (6)	0.25539 (12)	−0.1669 (4)	0.0515 (10)	
C4	−0.1490 (7)	0.26142 (13)	−0.2856 (4)	0.0638 (12)	
H4	−0.2087	0.2502	−0.3626	0.077*	
C5	0.0158 (6)	0.28417 (13)	−0.2892 (4)	0.0590 (11)	
H5	0.0674	0.2880	−0.3685	0.071*	
C6	0.1050 (5)	0.30142 (11)	−0.1744 (3)	0.0421 (9)	
C7	0.2344 (6)	0.40100 (11)	−0.1067 (4)	0.0450 (10)	
C8	0.0584 (6)	0.40745 (11)	−0.0611 (4)	0.0470 (10)	
H8	−0.0479	0.3923	−0.0935	0.056*	
C9	0.0417 (5)	0.43669 (11)	0.0336 (4)	0.0471 (10)	
H9	−0.0766	0.4412	0.0649	0.057*	
C10	0.2019 (5)	0.45956 (11)	0.0826 (4)	0.0429 (9)	
C11	0.3780 (5)	0.45208 (12)	0.0337 (4)	0.0534 (11)	
H11	0.4856	0.4670	0.0653	0.064*	
C12	0.3946 (6)	0.42264 (12)	−0.0617 (4)	0.0549 (11)	
H12	0.5119	0.4178	−0.0941	0.066*	
C13	0.1877 (5)	0.49231 (11)	0.1787 (4)	0.0463 (10)	
H13	0.2908	0.5098	0.1967	0.056*	
C14	0.0223 (5)	0.52968 (10)	0.3248 (3)	0.0373 (9)	
C15	0.1548 (5)	0.56282 (11)	0.3545 (3)	0.0384 (9)	
C16	−0.1307 (5)	0.53488 (11)	0.3982 (4)	0.0395 (9)	
C17	−0.3093 (5)	0.50989 (12)	0.3944 (4)	0.0570 (11)	
H17A	−0.4194	0.5264	0.3685	0.086*	
H17B	−0.3201	0.4988	0.4801	0.086*	
H17C	−0.3031	0.4884	0.3322	0.086*	
C18	−0.2542 (5)	0.59204 (12)	0.5268 (4)	0.0584 (12)	
H18A	−0.3351	0.6026	0.4530	0.088*	
H18B	−0.1979	0.6139	0.5788	0.088*	
H18C	−0.3297	0.5758	0.5796	0.088*	
C19	0.1734 (5)	0.61215 (11)	0.5422 (4)	0.0405 (9)	
C20	0.2802 (5)	0.64372 (12)	0.4972 (4)	0.0512 (10)	
H20	0.2788	0.6486	0.4075	0.061*	
C21	0.3887 (6)	0.66770 (13)	0.5880 (5)	0.0652 (12)	
H21	0.4630	0.6884	0.5586	0.078*	
C22	0.3878 (7)	0.66132 (14)	0.7211 (5)	0.0707 (13)	
H22	0.4591	0.6778	0.7814	0.085*	
C23	0.2799 (7)	0.63016 (14)	0.7642 (5)	0.0719 (13)	
H23	0.2789	0.6258	0.8540	0.086*	
C24	0.1737 (6)	0.60533 (12)	0.6761 (4)	0.0568 (11)	
H24	0.1028	0.5842	0.7063	0.068*	

### Atomic displacement parameters (Å^2^)

**Table:**

	*U*^11^	*U*^22^	*U*^33^	*U*^12^	*U*^13^	*U*^23^
Br1	0.0799 (4)	0.0750 (4)	0.0635 (3)	−0.0191 (3)	0.0040 (2)	0.0108 (2)
S1	0.0520 (7)	0.0551 (7)	0.0491 (7)	0.0086 (5)	0.0076 (5)	−0.0027 (5)
N1	0.0394 (19)	0.0448 (19)	0.044 (2)	0.0047 (14)	0.0013 (16)	0.0025 (15)
N2	0.0279 (17)	0.051 (2)	0.050 (2)	0.0043 (15)	0.0134 (14)	0.0036 (16)
N3	0.0293 (17)	0.048 (2)	0.048 (2)	−0.0008 (14)	0.0101 (15)	0.0016 (15)
O1	0.068 (2)	0.076 (2)	0.075 (2)	0.0053 (16)	0.0348 (17)	−0.0107 (16)
O2	0.0584 (18)	0.069 (2)	0.063 (2)	0.0112 (15)	−0.0128 (15)	0.0004 (15)
O3	0.080 (2)	0.0478 (17)	0.0394 (17)	0.0059 (14)	0.0052 (14)	0.0025 (13)
O4	0.0341 (15)	0.0641 (18)	0.0611 (19)	−0.0074 (13)	0.0166 (13)	−0.0066 (14)
C1	0.063 (3)	0.058 (3)	0.033 (2)	0.007 (2)	0.003 (2)	−0.0026 (18)
C2	0.072 (3)	0.071 (3)	0.033 (2)	−0.001 (2)	0.015 (2)	0.003 (2)
C3	0.056 (3)	0.050 (3)	0.048 (3)	0.008 (2)	0.000 (2)	0.0074 (19)
C4	0.077 (3)	0.076 (3)	0.037 (3)	−0.013 (3)	−0.003 (2)	−0.004 (2)
C5	0.069 (3)	0.075 (3)	0.033 (2)	−0.004 (2)	0.008 (2)	0.000 (2)
C6	0.049 (2)	0.044 (2)	0.033 (2)	0.0098 (18)	0.0056 (18)	0.0027 (17)
C7	0.054 (3)	0.040 (2)	0.041 (2)	0.002 (2)	0.008 (2)	0.0055 (18)
C8	0.047 (2)	0.053 (3)	0.041 (2)	−0.0049 (19)	−0.0005 (19)	0.0059 (19)
C9	0.046 (2)	0.050 (2)	0.047 (2)	0.006 (2)	0.0109 (19)	0.005 (2)
C10	0.043 (2)	0.040 (2)	0.046 (2)	0.0046 (18)	0.0061 (19)	0.0056 (18)
C11	0.044 (2)	0.054 (3)	0.064 (3)	−0.009 (2)	0.009 (2)	−0.006 (2)
C12	0.046 (3)	0.055 (3)	0.066 (3)	0.003 (2)	0.015 (2)	−0.001 (2)
C13	0.039 (2)	0.050 (2)	0.051 (3)	0.0012 (18)	0.007 (2)	0.0028 (19)
C14	0.030 (2)	0.041 (2)	0.041 (2)	0.0020 (17)	0.0004 (17)	0.0061 (17)
C15	0.030 (2)	0.046 (2)	0.039 (2)	0.0096 (18)	0.0053 (17)	0.0072 (17)
C16	0.029 (2)	0.042 (2)	0.047 (2)	0.0031 (17)	0.0022 (18)	0.0102 (18)
C17	0.037 (2)	0.063 (3)	0.072 (3)	−0.003 (2)	0.008 (2)	0.005 (2)
C18	0.038 (2)	0.075 (3)	0.065 (3)	0.011 (2)	0.018 (2)	0.002 (2)
C19	0.035 (2)	0.038 (2)	0.049 (3)	0.0077 (17)	0.0058 (18)	0.0037 (18)
C20	0.053 (3)	0.053 (3)	0.049 (3)	0.007 (2)	0.011 (2)	0.003 (2)
C21	0.064 (3)	0.054 (3)	0.078 (4)	−0.015 (2)	0.010 (3)	−0.004 (2)
C22	0.076 (3)	0.066 (3)	0.068 (4)	−0.017 (3)	−0.003 (3)	−0.014 (3)
C23	0.091 (4)	0.075 (3)	0.047 (3)	−0.007 (3)	−0.003 (3)	−0.002 (2)
C24	0.062 (3)	0.056 (3)	0.051 (3)	−0.006 (2)	0.003 (2)	0.007 (2)

### Geometric parameters (Å, °)

**Table:**

Br1—C3	1.900 (4)	C9—C10	1.403 (5)
S1—O1	1.425 (3)	C9—H9	0.9300
S1—O2	1.429 (3)	C10—C11	1.399 (5)
S1—O3	1.601 (3)	C10—C13	1.475 (5)
S1—C6	1.758 (4)	C11—C12	1.394 (5)
N1—C13	1.292 (4)	C11—H11	0.9300
N1—C14	1.401 (4)	C12—H12	0.9300
N2—C16	1.362 (4)	C13—H13	0.9300
N2—N3	1.416 (4)	C14—C16	1.380 (5)
N2—C18	1.474 (4)	C14—C15	1.451 (5)
N3—C15	1.418 (4)	C16—C17	1.497 (5)
N3—C19	1.433 (5)	C17—H17A	0.9600
O3—C7	1.430 (4)	C17—H17B	0.9600
O4—C15	1.239 (4)	C17—H17C	0.9600
C1—C2	1.380 (5)	C18—H18A	0.9600
C1—C6	1.398 (5)	C18—H18B	0.9600
C1—H1	0.9300	C18—H18C	0.9600
C2—C3	1.381 (5)	C19—C24	1.386 (5)
C2—H2	0.9300	C19—C20	1.394 (5)
C3—C4	1.390 (5)	C20—C21	1.387 (6)
C4—C5	1.382 (6)	C20—H20	0.9300
C4—H4	0.9300	C21—C22	1.378 (6)
C5—C6	1.393 (5)	C21—H21	0.9300
C5—H5	0.9300	C22—C23	1.380 (6)
C7—C12	1.370 (5)	C22—H22	0.9300
C7—C8	1.378 (5)	C23—C24	1.380 (6)
C8—C9	1.385 (5)	C23—H23	0.9300
C8—H8	0.9300	C24—H24	0.9300
			
O1—S1—O2	121.36 (18)	C10—C11—H11	119.5
O1—S1—O3	103.89 (16)	C7—C12—C11	118.5 (4)
O2—S1—O3	109.24 (15)	C7—C12—H12	120.7
O1—S1—C6	108.71 (18)	C11—C12—H12	120.7
O2—S1—C6	109.24 (17)	N1—C13—C10	121.7 (3)
O3—S1—C6	102.77 (16)	N1—C13—H13	119.2
C13—N1—C14	120.5 (3)	C10—C13—H13	119.2
C16—N2—N3	107.3 (3)	C16—C14—N1	123.0 (3)
C16—N2—C18	124.8 (3)	C16—C14—C15	107.8 (3)
N3—N2—C18	118.4 (3)	N1—C14—C15	129.2 (3)
N2—N3—C15	108.9 (3)	O4—C15—N3	123.2 (3)
N2—N3—C19	119.4 (3)	O4—C15—C14	131.9 (3)
C15—N3—C19	123.9 (3)	N3—C15—C14	104.9 (3)
C7—O3—S1	119.9 (2)	N2—C16—C14	110.5 (3)
C2—C1—C6	120.1 (4)	N2—C16—C17	121.9 (3)
C2—C1—H1	120.0	C14—C16—C17	127.5 (4)
C6—C1—H1	120.0	C16—C17—H17A	109.5
C1—C2—C3	120.4 (4)	C16—C17—H17B	109.5
C1—C2—H2	119.8	H17A—C17—H17B	109.5
C3—C2—H2	119.8	C16—C17—H17C	109.5
C2—C3—C4	120.0 (4)	H17A—C17—H17C	109.5
C2—C3—Br1	120.8 (3)	H17B—C17—H17C	109.5
C4—C3—Br1	119.1 (3)	N2—C18—H18A	109.5
C5—C4—C3	119.9 (4)	N2—C18—H18B	109.5
C5—C4—H4	120.0	H18A—C18—H18B	109.5
C3—C4—H4	120.0	N2—C18—H18C	109.5
C4—C5—C6	120.3 (4)	H18A—C18—H18C	109.5
C4—C5—H5	119.8	H18B—C18—H18C	109.5
C6—C5—H5	119.8	C24—C19—C20	120.2 (4)
C5—C6—C1	119.2 (4)	C24—C19—N3	121.5 (3)
C5—C6—S1	119.0 (3)	C20—C19—N3	118.2 (3)
C1—C6—S1	121.7 (3)	C21—C20—C19	119.1 (4)
C12—C7—C8	122.2 (4)	C21—C20—H20	120.5
C12—C7—O3	118.7 (3)	C19—C20—H20	120.5
C8—C7—O3	118.9 (4)	C22—C21—C20	120.9 (4)
C7—C8—C9	119.3 (4)	C22—C21—H21	119.5
C7—C8—H8	120.3	C20—C21—H21	119.5
C9—C8—H8	120.3	C21—C22—C23	119.3 (4)
C8—C9—C10	120.5 (4)	C21—C22—H22	120.4
C8—C9—H9	119.8	C23—C22—H22	120.4
C10—C9—H9	119.8	C24—C23—C22	121.1 (4)
C11—C10—C9	118.5 (4)	C24—C23—H23	119.5
C11—C10—C13	119.5 (3)	C22—C23—H23	119.5
C9—C10—C13	122.0 (3)	C23—C24—C19	119.4 (4)
C12—C11—C10	121.0 (4)	C23—C24—H24	120.3
C12—C11—H11	119.5	C19—C24—H24	120.3
			
C16—N2—N3—C15	−7.1 (4)	C10—C11—C12—C7	−0.1 (6)
C18—N2—N3—C15	−155.2 (3)	C14—N1—C13—C10	−177.2 (3)
C16—N2—N3—C19	−157.5 (3)	C11—C10—C13—N1	−171.6 (3)
C18—N2—N3—C19	54.4 (4)	C9—C10—C13—N1	11.5 (6)
O1—S1—O3—C7	−152.2 (3)	C13—N1—C14—C16	−175.3 (3)
O2—S1—O3—C7	−21.4 (3)	C13—N1—C14—C15	5.1 (6)
C6—S1—O3—C7	94.5 (3)	N2—N3—C15—O4	−174.2 (3)
C6—C1—C2—C3	−0.4 (6)	C19—N3—C15—O4	−25.4 (5)
C1—C2—C3—C4	−1.3 (6)	N2—N3—C15—C14	4.5 (4)
C1—C2—C3—Br1	176.6 (3)	C19—N3—C15—C14	153.3 (3)
C2—C3—C4—C5	1.8 (6)	C16—C14—C15—O4	178.2 (4)
Br1—C3—C4—C5	−176.2 (3)	N1—C14—C15—O4	−2.2 (6)
C3—C4—C5—C6	−0.6 (7)	C16—C14—C15—N3	−0.4 (4)
C4—C5—C6—C1	−1.1 (6)	N1—C14—C15—N3	179.2 (3)
C4—C5—C6—S1	177.1 (3)	N3—N2—C16—C14	6.9 (4)
C2—C1—C6—C5	1.6 (6)	C18—N2—C16—C14	152.4 (3)
C2—C1—C6—S1	−176.6 (3)	N3—N2—C16—C17	−172.9 (3)
O1—S1—C6—C5	−19.4 (4)	C18—N2—C16—C17	−27.4 (5)
O2—S1—C6—C5	−153.8 (3)	N1—C14—C16—N2	176.3 (3)
O3—S1—C6—C5	90.3 (3)	C15—C14—C16—N2	−4.0 (4)
O1—S1—C6—C1	158.8 (3)	N1—C14—C16—C17	−3.9 (6)
O2—S1—C6—C1	24.4 (4)	C15—C14—C16—C17	175.7 (3)
O3—S1—C6—C1	−91.5 (3)	N2—N3—C19—C24	29.2 (5)
S1—O3—C7—C12	86.2 (4)	C15—N3—C19—C24	−116.5 (4)
S1—O3—C7—C8	−99.0 (4)	N2—N3—C19—C20	−152.1 (3)
C12—C7—C8—C9	−0.1 (6)	C15—N3—C19—C20	62.2 (5)
O3—C7—C8—C9	−174.7 (3)	C24—C19—C20—C21	1.0 (5)
C7—C8—C9—C10	0.0 (6)	N3—C19—C20—C21	−177.7 (3)
C8—C9—C10—C11	0.0 (5)	C19—C20—C21—C22	−1.7 (6)
C8—C9—C10—C13	176.9 (3)	C20—C21—C22—C23	1.2 (7)
C9—C10—C11—C12	0.1 (6)	C21—C22—C23—C24	0.1 (7)
C13—C10—C11—C12	−177.0 (4)	C22—C23—C24—C19	−0.8 (7)
C8—C7—C12—C11	0.1 (6)	C20—C19—C24—C23	0.3 (6)
O3—C7—C12—C11	174.8 (3)	N3—C19—C24—C23	178.9 (4)

### Hydrogen-bond geometry (Å, °)

**Table:**

*D*—H···*A*	*D*—H	H···*A*	*D*···*A*	*D*—H···*A*
C17—H17A···O4^i^	0.96	2.40	3.361 (5)	176

Symmetry codes: (i) *x*−1, *y*, *z*.
